# Gut Microbiome in Psoriasis: An Updated Review

**DOI:** 10.3390/pathogens9060463

**Published:** 2020-06-12

**Authors:** Mariusz Sikora, Albert Stec, Magdalena Chrabaszcz, Aleksandra Knot, Anna Waskiel-Burnat, Adriana Rakowska, Malgorzata Olszewska, Lidia Rudnicka

**Affiliations:** 1 Department of Dermatology, Medical University of Warsaw, 02-091 Warsaw, Poland; chrabaszcz.magda@gmail.com (M.C.); waskiel.a@gmail.com (A.W.-B.); adriana.rakowska@gmail.com (A.R.); malgorzataolszewska@yahoo.com (M.O.); lidiarudnicka@gmail.com (L.R.); 2 Student Research Committee, Department of Dermatology, Medical University of Warsaw, 02-091 Warsaw, Poland; albertmstec@gmail.com (A.S.); ola.knot@gmail.com (A.K.)

**Keywords:** microbiome, psoriasis, gut-skin axis, gut barrier, systematic review

## Abstract

(1) Background: A growing body of evidence highlights that intestinal dysbiosis is associated with the development of psoriasis. The gut–skin axis is the novel concept of the interaction between skin diseases and microbiome through inflammatory mediators, metabolites and the intestinal barrier. The objective of this study was to synthesize current data on the gut microbial composition in psoriasis. (2) Methods: We conducted a systematic review of studies investigating intestinal microbiome in psoriasis, using the PRISMA checklist. We searched MEDLINE, EMBASE, and Web of Science databases for relevant published articles (2000–2020). (3) Results: All of the 10 retrieved studies reported alterations in the gut microbiome in patients with psoriasis. Eight studies assessed alpha- and beta-diversity. Four of them reported a lack of change in alpha-diversity, but all confirmed significant changes in beta-diversity. At the phylum-level, at least two or more studies reported a lower relative abundance of Bacteroidetes, and higher Firmicutes in psoriasis patients versus healthy controls. (4) Conclusions: There is a significant association between alterations in gut microbial composition and psoriasis; however, there is high heterogeneity between studies. More unified methodological standards in large-scale studies are needed to understand microbiota’s contribution to psoriasis pathogenesis and its modulation as a potential therapeutic strategy.

## 1. Introduction

Psoriasis is a common and chronic dermatological disease considered as a systemic inflammatory disorder [1]. Subclinical gut inflammation [2] and intestinal barrier dysfunction [3] reported in patients with psoriasis gave rise to the concept of the gut–skin axis. A growing body of evidence indicates that gut microbiota have a critical role in the regulation of metabolism [4], the immune system [5] and intestinal permeability [6]. The distortion in the biodiversity and composition of the gut microbiota, known as gut dysbiosis, has been linked to metabolic syndrome [7], inflammatory arthritis [8], depression [9], cardiovascular disease [10], inflammatory bowel diseases [11], which all are psoriasis comorbidities. Preclinical investigations provide evidence for the role of the gut microbiome in psoriasis pathogenesis. In mice with an experimental model of psoriasis induced by imiquimod, oral treatment with broad-spectrum antibiotic reduces the severity of skin inflammation through downregulation of Th17 immune response [12,13]. These results are supported by clinical observations based on a case series showing improvement in psoriatic skin lesions after antibiotic treatment [14], modulation of gut microbiota by probiotics [15] or fecal microbial transplantation [16].

In recent years, advancements in next-generation sequencing technologies have led to a thorough exploration of the intestinal microbiota composition [17]. However, the association of psoriasis with gut dysbiosis is mainly based on limited studies with small number of patients involved. Thus, the study aims to conduct an up-to-date systematic review of available gut microbiome composition studies in psoriasis, identify current methodological inconsistencies and outline directions for future research.

## 2. Materials and Methods

The systematic review was performed according the Preferred Reporting Items for Systematic Reviews and Meta-Analyses (PRISMA) guidelines [18]. The primary outcome measure of our study was a comparison of relative abundances of bacterial phyla, families, and genera in the gut microbiome of patients with psoriasis. Discrepancies between investigators at every stage of review were thoroughly discussed by all authors until a consensus was reached.

### 2.1. Search Strategy

Studies were identified by searching electronic databases and scanning reference lists of articles. We searched four electronic databases from their inception to March 2020: MEDLINE (via PubMed), Embase (via OvidSP), Web of Science Core Collection, and Scopus. To identify studies comparing gut microbiome composition in patients with psoriasis and normal healthy controls the combination of the following keywords was included: “psoriasis”, “psoriatic arthritis”, “microbiome”, “microbiota”, “bacteria”, “dysbiosis”, “gut”, “gastrointestinal”, “intestine”, “stool”, “fecal”. Reference lists of the retrieved articles and relevant reviews were manually searched to identify further manuscripts not captured by the electronic searches.

### 2.2. Inclusion/Exclusion Criteria

The eligible inclusion criteria were presented as follows:

1. Subjects aged 18 years or older;
2. Human case-control studies investigating the association between gut microbiota and psoriasis
3. usage of culture-independent, high-throughput sequencing methods for gut microbiota quantification;
4. Articles published in English.

We excluded from the analysis: review papers, conference abstracts, case reports, expert opinions, editorials, and studies using animal models.

### 2.3. Study Selection

Four authors (M.S., A.S., M.C., A.K.) independently reviewed the titles and abstracts of all papers retrieved by the search strategy. Relevant full-text articles were evaluated for fulfilling the inclusion and exclusion criteria.

### 2.4. Quality Assessment

The quality of included studies was rated according to the Newcastle–Ottawa Scale (NOS), which is a valid tool for quality assessment of case–control studies. The scale evaluates three categories:

1. Selection (adequate case definition, representativeness of the cases, selection of controls, definition of controls);
2. Comparability (factors that the study controlled for by design or analysis to improve the comparability of baseline characteristics of cases and controls);
3. Exposure (ascertainment of exposure, same method of ascertainment for cases and controls, nonresponse rate).

### 2.5. Data Extraction

Six authors (M.S., A.S., M.C., A.K., A.W.-B., A.R.) in a pair-wise manner extracted the following data from the eligible studies: first author, publication year, country where the study was conducted, participant characteristics (sample size, age, sex, current treatment), sample materials, DNA extraction method, 16S rRNA variable region, sequencing platform, data analysis platform, reference sequences database employed in the studies, microbial diversity, alterations in gut microbiota in patients with psoriasis.

## 3. Results

### 3.1. Search Results and Study Characteristics

**Figure 1:**
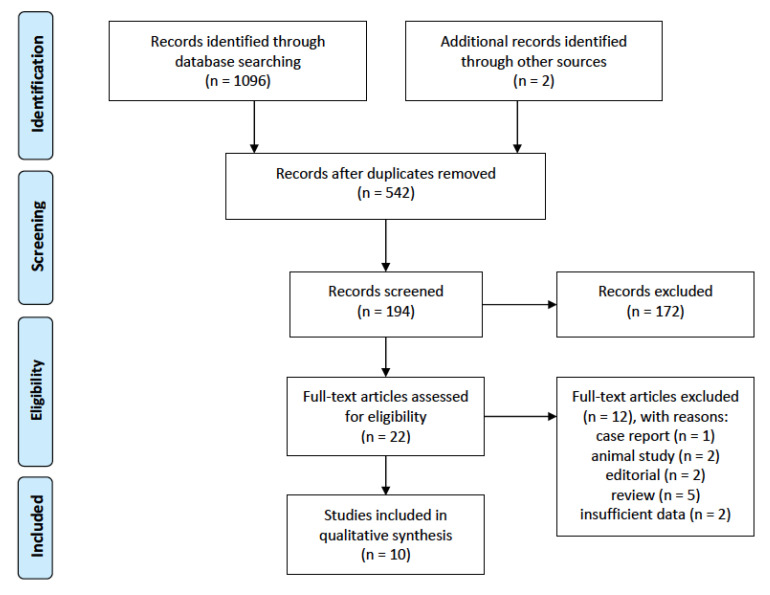
PRISMA flow diagram of study selection for inclusion in the systematic review; PRISMA—Preferred Reporting Items for Systematic Reviews and Meta-Analyses.

Ten records were included for the final analysis. The details of the article selection process are shown in Figure 1.

All eligible studies were published between 2015 and 2019 [19,20,21,22,23,24,25,26,27,28]. The general characteristics of the included studies are provided in Table 1. The studies were conducted in seven countries: China (two studies), Spain (two studies), Taiwan (two studies), Israel (one study), Netherland (one study), Egypt (one study), and the US (one study). The total sample size of included studies was 431 (299 patients with psoriasis and 254 healthy controls). Codoner et al. [24] compared psoriatic patients with a cohort of over 300 healthy individuals extracted from the Human Microbiome Project. This study was excluded from the age and sex analysis because the age range as well as gender distribution data for control group were not reported. The remaining studies were sex- and age-matched. The mean age was similar between compared groups and varied from 39.4 to 52.7 in patients with psoriasis and 40.4 to 52.9 in controls. Male patients constituted 56.6% of the studied population (57.5% in psoriasis group and 55.5% in control group).

All of the included studies used stool samples for gut microbiota profiling (sample and methodology characteristics of the included studies are summarized in Table 2). In five studies, the samples were frozen immediately after collection and remained frozen until DNA extraction. Other studies shipped the samples on ice (n = 3), and used either a nucleotide stabilizer before freezing (n = 1) or shipped the samples at ambient temperature within 48 h (n = 1). The majority of studies used different DNA extraction kits with QIAamp DNA Stool Mini Kit (Qiagen, Hilden, Germany) and PowerSoil DNA Extraction Kits (MoBio, Carlsbad, CA) used in several studies: five and two, respectively. 

Eight studies have employed 16S rRNA gene sequencing, although with different variable regions for DNA amplification: region V1–V2 [28], region V2–V3 [22], region V3–V4 [19,23,24], region V4 [20,25], region V4–V5 [21]. Eppinga et al. [26] compared the abundance of *Faecalibacterium prausnitzii* and *Escherichia coli* in the fecal samples using quantitative PCR, while Masallat et al. [27] analyzed abundance of *Firmicutes*, *Bacteroidetes* and *Actinobacteria*. The most commonly used sequencing platform was the Illumina MiSeq platform. Data analysis pipelines were QIIME, UPARSE and USEARCH. Reference sequence databases used in the studies included the Silva database, Greengenes database, and Ribosomal Database Project.

#### 3.1.1. Gut Microbiome Diversity in Psoriasis

Eight of ten studies assessed alpha-diversity. The majority of them fail to show significant changes [19,20,23,25], two reported lower alpha-diversity [22,28] and one greater diversity in psoriasis [24]. Huang et al. demonstrated lower community richness in psoriasis with similar level of diversity [21]. All studies used Shannon’s Diversity Index to assess alpha-diversity. Four studies additionally assess alpha-diversity with Simpson or Chao1; two studies used the abundance-based Coverage Estimator (ACE) and Faith phylogenetic diversity index. Observed species richness and rarefaction curve were also mentioned.

Beta-diversity was assessed in all studies that performed 16S rRNA gene sequencing, all of which reported a significant difference between psoriasis and healthy controls. However, a study by Chen et al. [23] reported that differences reached statistical significance only for psoriatic patients with a BMI lower than 25. Employed estimates of beta-diversity included Bray–Curtis dissimilarity distance, principal coordinate analysis, and weighted and unweighted UniFrac distances. An overview of alpha- and beta-diversity in psoriasis is provided in Table 3.

#### 3.1.2. Gut Microbiome Alterations in Psoriasis

All studies confirmed the association of psoriasis and gut microbiota dysbiosis. At the phylum level, *Bacteroidetes* had a lower relative abundance and *Firmicutes* a higher relative abundance in patients with psoriasis than in healthy controls [20,22,23]. However, in a study by Huang et al., *Bacteroidetes* was reported to be increased and *Firmicutes* decreased in psoriasis [21]. Additionally, two studies showed decreased amounts of *Proteobacteria* in psoriatic cohort [20,22]. Studies for *Actinobacteria* provide conflicting results—an increase in two studies [20,22] and decrease in the other two studies [27,28]. 

At the family level, the relative abundance of *Ruminococcaceae*, *Lachnospiraceae*, *Clostridiales Family XIII*, *Peptostreptococcaceae*, *Enterococcaceae*, *Coriobacteriaceae*, and *Eggerthellaceae* was increased in psoriasis, whereas *Prevotellaceae*, *Barnesiellaceae*, *Tannerellaceae*, *Rikenellaceae*, *Porphyromonadaceae*, *Marinifilaceae*, *S24-7*, *Lactobacillaceae*, *Streptococcaceae*, *Pasteurellaceae*, *Burkholderiaceae*, *Desulfovibrionaceae*, *Victivallaceae*, and *Verrucomicrobiaceae* decreased. There were conflicting results for *Bacteroidaceae*, *Erysipelotrichaceae*, *Veillonellaceae* and Bifidobacteriaceae. Some studies reported these families to be decreased in psoriasis [22,23,28] while others showed their increase [22,25]. 

At the genus level, *Paraprevotella*, *Barnesiella*, *Alistipes*, *Allobaculum*, *Coprobacillus*, *Carnobacterium*, *Granulicatella*, *Rothia*, *Gordonibacter*, *Thermus* were found to be decreased. Following genera were relatively increased in psoriasis: *Ruminococcus*, *Subdoligranulum*, *Blautia*, *Coprococcus*, *Dorea*, *Christensenella*, *Streptococcus*, *Lactococcus*, *Enterococcus*, *Bacillus*, *Collinsella*, *Slackia*. Divergent findings were reported for *Bacteroides*, *Parabacteroides*, *Faecalibacterium*, *Lachnospira*, *Akkermansia*, *Sutterella*, and *Bifidobacterium*.

At the species level, *Prevotella copri*, *Faecalibacterium prausnitzii* and *Akkermiansia muciniphila* were found to be significantly decreased, while *Ruminococcus gnavus*, *Dorea formicigenerans*, *Clostridium citroniae*, *Escherichia coli*, and *Collinsella aerofaciens* were increased in patients with psoriasis compared to control group. However, these alterations have not been confirmed in more than one study. Details indicating the altered bacteria are shown in Table 4.

#### 3.1.3. Changes in Gut Microbiota after Antipsoriatic Treatment

To date, only one study compared changes in the composition of the pre-treatment and post-treatment intestinal microbiome. Yeh et al. [19] analyzed stool samples from 12 healthy individuals and 34 psoriatic patients at baseline, 3 and 6 months after biological treatment with secukinumab (24 patients) or ustekinumab (10 patients). Secukinumab treatment caused more profound alterations in gut microbiome, such as an increase in the relative abundance of phylum *Proteobacteria* and decrease in *Bacteroidetes* and *Firmicutes*. At other levels of taxonomic classification, treatment with secukinumab led to an increase in *Citrobacter* and a decrease in *Aeromonas*, *Bacteroides*, *Ruminococcus torques* at the genera level. At the family level, *Enterobacteriaceae* and *Pseudomonadaceae* were significantly increased, while *Aeromonadaceae* decreased. In contrast, there was no significant change in gut microbiome after ustekinumab treatment, where only genus *Coprococcus* significantly increased after 6 months.

#### 3.1.4. Quality of the Evidence

All included studies were assessed for quality according to NOS and scored between 4 to 8 points (Table 1).

## 4. Discussion

Recent advances in genome sequencing and bioinformatic analysis have enabled a greater understanding of the complex host–microbiome associations. However, since the first publication on gut microbiome differences in psoriasis by Scher et al. [28], there have been more opinion papers and editorials on the significance of the gut–skin axis than laboratory investigations. This paper provides a detailed and comprehensive systematic review regarding gut microbiome in patients with psoriasis. To cover a wider range of microbiome alterations we extracted the data of every available bacterial group using the lowest taxonomic level of each included study. 

In this review, we found that most of the studies reported no significant differences in alpha-diversity between patients with psoriasis and healthy subjects based on similar indices. Comparable findings have also been shown in other chronic inflammatory diseases, namely multiple sclerosis [29], ankylosing spondylitis [30] and ulcerative colitis [31]. Although alpha-diversity remained unchanged in psoriasis, all studies revealed significant beta-diversity differences. Diversity indices are one of the tools enable to characterized microbiome [32]. Alpha-diversity describes the variety of the microbial community in a single sample, taking into account the number of different taxa and their relative abundances. Beta-diversity measures diversity of microbial communities’ composition between different samples. Microbial community may experience a complete shift in composition, where no taxa are shared, but can still have similar alpha-diversity index [33]. Given all the above findings, it can be speculated that gut dysbiosis in psoriasis may be a result of differential abundance of bacteria instead of the quantity of bacterial species. 

We presented several taxa that differed in their relative abundance in psoriasis. For instance, a reduction in *Bacteroides* and *Proteobacteria* with increased proportions of *Firmicutes* and *Actinobacteria*, at phylum level was reported in more than one study. These four phyla constitute > 98% of the gut microbiota. Therefore, the *Firmicutes/Bacteroidetes* (F/B) ratio is considered as an important marker of gut microbiota state. Several studies have shown that an altered F/B ratio in gut microbiome is associated with psoriasis comorbidities, such as cardiovascular diseases [34], obesity [35], insulin resistance [36] and nonalcoholic fatty liver disease [37]. Only one study by Huang et al. reported opposite changes in *Bacteroides* and *Firmicutes* [21]. This dissimilarity partially may be related to a small number of participants and very diverse group, which consisted of people with plaque psoriasis, pustular psoriasis, erythrodermic psoriasis and psoriatic arthritis.

The tendencies for the change of *Firmicutes* and *Bacteroidetes* in patients with psoriasis are also present at the lower taxonomic levels. Families *Bacteroidaceae* and *Prevotellaceae* are two important subgroups in phylum *Bacteroides*. While alterations in *Bacteroidaceae* and *Bacteroides* have been confirmed in psoriasis, there is no agreement about the direction of these changes. Similarly, the role of these bacteria is also controversial. *Bacteroides fragilis*, can produce enterotoxins responsible for inflammation and impairment of the intestinal barrier [38]. Conversely, non-toxigenic *Bacteroides fragilis* exerts beneficial effects by production of short-chain fatty acids and polysaccharide A with anti-inflammatory properties [38]. *Prevotella copri*, another species belonging to the same phylum, was found to be decreased in psoriasis. This finding is in opposite to other inflammatory diseases—ankylosing spondylitis [39] or rheumatoid arthritis [40]—where there is an increased abundance of *P. copri*. This shift in microbial composition may be modulated by the duration of the disease as well as by using conventional or biological therapy [39,40]. A problem that remains to be resolved is whether *P. copri* is beneficial or detrimental, as studies report conflicting results on metabolic and inflammatory actions [41]. The pro- or anti-inflammatory effect may be dependent on the diet and the fact that *P. copri* is not a monotypic species but composed of four distinct clades [42]. 

Regarding the second important phylum, *Firmicutes*, at least two studies have found increased abundance of families *Ruminococcaceae* and *Lachnospiraceae*, with depletion of *Faecalibacterium prausnitzii* and augmentation of *Ruminococcus gnavus* at the species level. *F. prausnitzii* metabolites exert a protective effect on the gut barrier and inhibit the activation of the NF-κB, altering the pro-inflammatory response [43]. The depletion of *F. prausnitzii* has been associated with inflammatory disorders, such as inflammatory bowel disease or ankylosing spondylitis [26,39]. On the other hand, *R. gnavus* produces an inflammatory polysaccharide and contributes to gut barrier dysfunction. Its increased abundance was observed in inflammatory bowel disease, spondyloarthritis, eczema and coronary artery disease [44].

It is still not clear whether psoriasis is an effect or a cause of the observed disbalance between beneficial and pathogenic microbes. These complex gut microbiome–host interactions include destruction of tight junction proteins and intestinal barrier integrity, initiation and maintenance of inflammation, and changes in metabolites production [3,45]. Another proposed mechanism underlying the link between gut dysbiosis and skin changes implies the modulation of T cells differentiation and function with an imbalance between Th17 and T regulatory (Treg) cells. In experimental model of psoriasis, it has been shown that alterations in the intestinal microbiota may promote Th17-mediated skin inflammation [12,13]. In this context, the study by Yeh et al. [19] provides very interesting results, showing significantly greater changes in the gut microbiome of patients with psoriasis treated with secukinumab (interleukin-17 inhibitor) compared to ustekinumab (inhibitor of interleukin-12 and -23). On the other hand, in the same study, the baseline composition of the microbiome differed significantly between responders and non-responders to secukinumab treatment [19]. In complex interactions in the gut–skin axis, psoriasis severity should also be considered in addition to the microbiome and intestinal barrier. It has been shown that Psoriasis Activity and Severity Index (PASI) positively correlates with the blood concentration of intestinal barrier damage biomarkers [46,47]. Much less is known about the potential relationship between the composition of gut microbiome and the severity of psoriasis. Among the analyzed studies, only two assessed such a potential relationship. Masallat et al. [27] found positive correlation between PASI and *Firmicutes/Bacteroidetes* ratio as well as a negative correlation between PASI and *Actinobacteria* phylum. On the contrary, according to a study by Chen et al. [23], disease activity assessed by PASI score did not significantly affect the abundance profile of gut microbiome among patients with psoriasis.

This review clearly demonstrated that despite significant differences in taxa between psoriasis and control groups, there is a lack of consistency in the results concerning microbial diversity, relative abundance, or directionality of differences. The results’ heterogeneity may be explained, to a certain degree, by differences in study population, design and methodology. Psoriasis is a heterogeneous disorder with a broad spectrum of clinical presentations. Similar to other chronic inflammatory diseases, disease activity, disease duration, comorbidities and treatment, may be also responsible for microbiota changes [30,31,39]. The low sample size, ranging from 14 to 52 patients with psoriasis, makes it impossible to assess the above factors for their confounding influence on microbiome composition. Unlike the fixed and unchangeable human genome, the gut microbiome is highly dynamic and it can be modulated by age, sex, geographical background and diet [32,48]. None of the analyzed studies were multicenter, which may account for inter-study differences as seven countries were represented. Another mentioned variable is diet, but most studies did not examine dietary habits. For future studies, it is highly recommended to provide a complete assessment of nutrient intake by food diaries or food frequency questionnaires.

We found different methodologies in all phases, starting from sample collection transportation and storage, through DNA extraction and sequencing to computational analysis. Microbiota profiling requires particular care in terms of methodology choices, while each step can be a potential source of bias [49]. All the included studies investigated the gut microbiome using fecal samples. This approach carries a serious limitation; it does not allow to determine changes in the microbiome across the intestine. There is also difference in composition of luminal- and mucosal-associated microbiota [50]. Additionally, it may not be clear whether an identified taxon maintains pathogenic role or is just nonviable and transient. To reduce this bias, there is a need for the simultaneous application of several sampling techniques, such as intestine bioptates, mucosal–luminal interface aspirates or colonic lavage. 16S rRNA gene profiling is the most common approach to the study of a microbiome. Only two articles limited their search of the gut microbiome a priori to specific taxa: *F. prausnitzii* and *E. coli* [26] or *Firmicutes*, *Bacteroidetes* and *Actinobacteria* [27]. Even among the eight studies that employed 16S rRNA gene sequencing, different selection of targeted variable region will bias identification and quantification [51]. 

## 5. Conclusions

Several studies demonstrate differences in the microbiome composition of patients with psoriasis. However, no specific changes in results, methodological inconsistencies and unaddressed confounders make the translation into clinical practice difficult. Useful information may be obtained from a meta-analysis, but, currently, due to the studies’ high heterogeneity and limited number of publications, this approach seems to be impractical. Instead, we highlighted commonalities and identify existing problems. An in-depth understanding of the complex gut–skin axis requires the standardization of study protocols and a unified methodological approach. Finally, large-scale, longitudinal studies with a multiomics assessment would give detailed insight into the microbiome–host interactions and allow to identify targeted therapies.

## Figures and Tables

**Table 1:** General characteristic of the included studies.

Study	Country	Case Number	Case Age (Mean ± SD)	Case Gender Men, (%)	Antipsoriatic Therapy	Control Number	Control Age (Mean ± SD)	Control Gender Men, (%)	NOS
Yeh et al., 2019 [19]	Taiwan	34	SEC: 51 ± 12 UST: 48.4 ± 12.7	25 (73.5%)	No treatment except for topical agents *	12	48.4 ± 13.3	10 (83.3%)	8
Shapiro et al., 2019 [20]	Israel	24	52.7 ± 11.6	16 (66.7%)	Topical treatment (22) Biologics (2)	22	43.9 ± 12.7	16 (72.7%)	8
Huang et al., 2019 [21]	China	35	52.1 ± 3.0	22 (62.9%)	NR	27	52.9 ± 1.5	16 (59.3%)	6
Hidalgo-Cantabrana et al., 2019 [22]	Spain	19	49 ± 11	12 (63.2%)	No treatment except for topical corticosteroids	20	43 ± 11	5 (25%)	6
Chen et al., 2018 [23]	Taiwan	32	42.8 ± 12.6	25 (78.1%)	Phototherapy (8) DMARDs/ Biologics (20)	64	44.2±10.8	50 (78.1%)	7
Codoner et al., 2018 [24]	Spain	52	41.2 ± 14.4	25 (48.1%)	NR	NR	NR	NR	4
Tan et al., 2018 [25]	China	14	47.5 ± 4.7	10 (71.4%)	NR	14	40.4 ± 2.5	8 (57.1%)	7
Eppinga et al., 2016 [26]	Netherlands	29	46 ± 14.0	12 (41.4%)	No treatment (27) DMARDs (2)	33	41 ± 14.9	10 (30.3%)	6
Massallat et al., 2016 [27]	Egypt	45	42.3 ± 10	18 (40%)	NR	45	44.2 ± 7.1	20 (44.4%)	8
Scher et al., 2015 [28]	US	15	39.4	7 (46.7%)	No systemic treatment	17	42.2	6 (35.3%)	6

SD—standard deviation; NOS—the Newcastle-Ottawa Scale; NR—not reported; DMARDs—disease-modifying antirheumatic drugs; *—microbiome analysis before biological treatment.

**Table 2:** Sample and methodology characteristics of the included studies.

Study	Sample	Sample Transport	DNA Extraction	Microbiota Analysis Technique	Sequencing Target	Sequencing Platform	Data Analysis Platform	Reference Sequences Database
Yeh et al., 2019 [19]	stool	DNA stabilizer, Immediate freezing, Transport on ice	QIAamp DNA Stool Mini Kit	16S rRNA gene sequencing	V3–V4	Ilumnia MiSeq platform	QIIME	Kyoto Encyclopedia of Genes and Genomies database
Shapiro et al., 2019 [20]	stool	Immediate freezing	PowerSoil HTP 96 Kit	16S rRNA gene sequencing	V4	Ilumnia MiSeq platform	QIIME	Greengenes database
Huang et al., 2019 [21]	stool	Transport on ice	PowerSoil HTP 96 Kit	16S rRNA gene sequencing	V4–V5	Ilumnia MiSeq platform	UPARSE	National Center for Biotechnology Information Sequence Read Archive database
Hidalgo-Cantabrana et al., 2019 [22]	stool	Immediate freezing	QIAamp DNA Stool Mini Kit	16S rRNA gene sequencing	V2–V3	Ion Gene Studio S5 sequencer	QIIME	SILVA database
Chen et al., 2018 [23]	stool	Transport on ice	QIAamp DNA Stool Mini Kit	16S rRNA gene sequencing	V3–V4	Ilumnia MiSeq platform	UPARSE	Greengenes database
Codoner et al., 2018 [24]	stool	Immediate freezing	QIAamp DNA Stool Mini Kit	16S rRNA gene sequencing	V3–V4	Ilumnia MiSeq platform	QIIME	National Center for Biotechnology Information
Tan et al., 2018 [25]	stool	Immediate freezing	E.Z.N.A. stool DNA Kit	16S rRNA gene sequencing	V4	Ilumnia MiSeq platform	USEARCH	Ribosomal Database Project (RDP)
Eppinga et al., 2016 [26]	stool	Ambient temperature	NR	real-time quantitative PCR	-	quantitative PCR	-	-
Massallat et al., 2016 [27]	stool	NR	QIAamp DNA Stool Mini Kit	real-time quantitative PCR	-	quantitative PCR	-	-
Scher et al., 2015 [28]	stool	NR	NR	16S rRNA gene pyro-sequencing	V1–V2	454 GS FLX Titanium platform	Mothur	SILVA database

NR—not reported.

**Table 3:** Alpha- and beta-diversity of gut microbiome in psoriasis compared with healthy controls.

Study	Indices of α-Diversity	Gut Microbiota α-Diversity in Psoriasis	Indices of β-Diversity	Gut Microbiota β-Diversity in Psoriasis
Yeh et al. [19]	Shannon index, Simpson index	No differences were observed	UniFrac analysis (weighted and unweighted analyses), Bray Curtis index	Significant difference
Shapiro et al. [20]	Shannon index, rarefaction curves	No differences were observed	UniFrac analysis (weighted and unweighted analyses)	Significant difference
Huang et al. [21]	Shannon index, Simpson index, ACE index, Chao1 index	Shannon and Simpson indexes—no differences ACE and Chao indexes—decreased in psoriasis	PCA based on the Bray-Curtis dissimilarity distance	Significant difference
Hidalgo-Cantabrana et al. [22]	Shannon index, Chao1 index, Faith’s phylogenetic diversity index	Lower diversity in psoriasis	unweighted Unifrac analysis	Significant difference
Chen et al. [23]	Shannon index, Simpson index, Chao1 index, number of observed OTUs	No differences were observed	UniFrac analysis (weighted and unweighted analyses), Bray Curtis index	Significant difference (psoriasis patients with BMI < 25)
Codoner et al. [24]	Shannon index	Greater diversity in psoriasis	PCA	Significant difference
Tan et al. [25]	Shannon index, Simpson index, ACE index, Chao1 index	No differences were observed	PCA, UPGMA	Significant difference
Scher et al. [28]	Shannon index, Faith’s phylogenetic diversity index	Lower diversity in psoriasis	unweighted Unifrac analysis	Significant difference

ACE—abundance-based coverage estimator; OTUs—operational taxonomic units; PCA—principal component analysis; UPGMA—The Unweighted Pair Group Method with Arithmetic Mean.

**Table 4:** Gut microbiota alterations in psoriasis.

Phylum	Class	Order	Family	Genus	Species
*Bacteroidetes* ↑ [21] ↓ [20,22,23]	*Bacteroidia*	*Bacteroidales*	*Bacteroidaceae* ↑ [25] ↓ [22,23]	*Bacteroides* ↑ [25] ↓ [22,24]	
			*Prevotellaceae* ↓ [22,23]	*Prevotella*	*Prevotella copri* ↓ [20]
				*Paraprevotella* ↓ [20,22]	
			*Barnesiellaceae* ↓ [22]	*Barnesiella* ↓ [22]	
			*Tannerellaceae* ↓ [22]		
			*Rikenellaceae* ↓ [22]	*Alistipes* ↓ [22]	
			*Tannerellaceae*	*Parabacteroides* ↑ [21] ↓ [22,28]	
			*Porphyromonadaceae* ↓ [28]		
			*S24-7* ↓ [25]		
		*Marinilabiliales*	*Marinifilaceae* ↓ [22]		
*Firmicutes* ↑ [20,22,23] ↓ [21]	*Clostridia*	*Clostridiales*	*Ruminococcaceae* ↑ [22,23]	*Faecalibacterium* ↑ [20,24] ↓ [22]	*Faecalibacterium prausnitzii* ↓ [26]
				*Ruminococcus* ↑ [20,22]	*Ruminococcus gnavus* ↑ [20]
				*Subdoligranulum* ↑ [22]	
			*Lachnospiraceae* ↑ [22,23]	*Lachnospira* ↑ [21] ↓ [20]	
				*Blautia* ↑ [20,22]	
				*Coprococcus* ↑ [20]	
				*Dorea* ↑ [20]	*Dorea formicigenerans* ↑ [20]
			*Clostridiales Family XIII* ↑ [22]		
			*Peptostreptococcaceae* ↑ [22]		
			*Christensenellaceae*	*Christensenella* ↑ [20]	
			*Clostridiaceae*	*Clostridium*	*Clostridium citroniae* ↑ [25]
	*Erysipelotrichia*	*Erysipelotrichales* ↓ [28]	*Erysipelotrichaceae* ↑ [22] ↓ [28]	*Allobaculum* ↓ [22]	
				*Coprobacillus* ↓ [28]	
	*Bacilli*	*Lactobacillales*	*Lactobacillaceae* ↓ [22]		
			*Streptococcaceae* ↓ [22]	*Streptococcus* ↑ [21]	
				*Lactococcus* ↑ [21]	
			*Carnobacteriaceae*	*Carnobacterium* ↓ [21]	
				*Granulicatella* ↓ [21]	
			*Enterococcaceae* ↑ [25]	*Enterococcus* ↑ [25]	
		*Bacillales*	*Bacillaceae*	*Bacillus* ↑ [21]	
	*Negativicutes*	*Veillonellales*	*Veillonellaceae* ↑ [25] ↓ [22]		
*Verrucomicrobia* ↓ [25]	*Verrucomicrobiae* ↓ [25]	*Verrucomicrobiales* ↓ [25]	*Akkermansiaceae*	*Akkermansia* ↑ [24] ↓ [25]	*Akkermiansia muciniphila* ↓ [25]
			*Verrucomicrobiaceae*↓ [25]		
*Proteobacteria* ↓ [20,22]	*Gammaproteobacteria*	*Enterobacterales*	*Enterobacteriaceae*	*Escherichia*	*Escherichia coli* ↑ [26]
		*Pasteurellales*	*Pasteurellaceae* ↓ [22]		
	*Betaproteobacteria*	*Burkholderiales*	*Burkholderiaceae* ↓ [22]		
			*Sutterellaceae*	*Sutterella* ↑ [21] ↓ [20]	
	*Deltaproteobacteria*	*Desulfovibrionales*	*Desulfovibrionaceae* ↓ [22]		
*Actinobacteria* ↑ [20,22] ↓ [27,28]	*Actinobacteria* ↓ [28]	*Bifidobacteriales* ↓ [28]	*Bifidobacteriaceae* ↑ [22] ↓ [28]	*Bifidobacterium* ↑ [20,22] ↓ [28]	
		*Micrococcales*	*Micrococcaceae*	*Rothia* ↓ [21]	
	*Coriobacteriia*	*Coriobacteriales*	*Coriobacteriaceae* ↑ [22]	*Collinsella* ↑ [20,22]	*Collinsella aerofaciens* ↑ [20]
		*Eggerthellales*	*Eggerthellaceae* ↑ [22]	*Slackia* ↑ [22]	
				*Gordonibacter* ↓ [21]	
*Lentisphaerae*	*Lentisphaeria*	*Victivallales*	*Victivallaceae* ↓ [22]		
*Deinococcus–Thermus*	*Deinococci*	*Thermales*	*Thermaceae*	*Thermus* ↓ [21]	
*Tenericutes* ↓ [25]	*Mollicutes* ↓ [25]				
